# Identification of novel COX-2 / CYP19A1 axis involved in the mesothelioma pathogenesis opens new therapeutic opportunities

**DOI:** 10.1186/s13046-021-02050-1

**Published:** 2021-08-17

**Authors:** Barbara Nuvoli, Barbara Antoniani, Roberta Libener, Antonio Maconi, Andrea Sacconi, Mariantonia Carosi, Rossella Galati

**Affiliations:** 1 Preclinical Models and New Therapeutic Agents Unit, IRCCS Regina Elena National Cancer Institute, Rome, Italy; 2 Anatomy Pathology Unit, IRCCS Regina Elena National Cancer Institute, Rome, Italy; 3 Department of Integrated Activities Research and Innovation, SS Antonio and Biagio General Hospital, Alessandria, Italy; 4 Clinical Trial Center, Biostatistics and Bioinformatics Unit, IRCCS Regina Elena National Cancer Institute, Rome, Italy

**Keywords:** Malignant Pleural Mesothelioma, Inflammation, Cyclooxygenase-2, Aromatase

## Abstract

**Background:**

Based on previous studies highlighting that the induction of cyclooxygenase-2 (COX-2) and high prostaglandin E2 (PGE2) levels contribute to the pathogenesis of malignant pleural mesothelioma (MPM), and that aromatase (CYP19A1), an enzyme that plays a key role in estrogen biosynthesis, along with estradiol (E2) were expressed in MPM, this study aimed to investigate the possible interplay between COX-2 and CYP19A1 in the pathogenesis of mesothelioma, as well as the underlying mechanism.

**Methods:**

The interaction between COX-2 and CYP19A1 was first investigated on different MPM lines upon PGE2, and COX-2 inhibitor (rofecoxib) treatment by western blot, RT-PCR. The key regulatory pathways involved in the COX-2 and CYP19A1 axis were further studied in MPM cells, after rofecoxib and exemestane (CYP19A1 inhibitor) treatment in monotherapy and in combination, by cell cycle distribution, western blot, and combination index analysis. To explore the role of COX-2/CYP19A1 axis in 3D preclinical models of MPM cells, we analyzed the effect of combination of COX-2 and CYP19A1 inhibitors in mesosphere formation. Immunohistochemical analysis of MPM mesosphere and specimens was utilized to evaluate the involvement of COX-2 on the CYP19A1 activity and the relationship between E2 and COX-2.

**Results:**

PGE2 or rofecoxib treatment caused in MPM cells an increased or decreased, respectively, CYP19A1 expression at mRNA and protein levels. The effect of rofecoxib and exemestane combination in MPM cell proliferation was synergistic. Activation of caspase-3 and cleavage of PARP confirmed an apoptotic death for MPM cell lines. Increased expression levels of p53, p21, and p27, downregulation of cyclin D1 and inhibition of Akt activation (pAKT) were also found. The antagonistic effect of rofecoxib and exemestane combination found only in one cell line, was reverted by pretreatment with MK2206, a pAKT inhibitor, indicating pAKT as an actionable mediator in the COX-2-CYP19A1 axis. Reduction of size and sphere-forming efficiency in MPM spheres after treatment with both inhibitor and a decrease in COX-2 and E2 staining was found. Moreover, immunohistochemical analysis of 46 MPM samples showed a significant positive correlation between COX-2 and E2.

**Conclusions:**

Collectively, the results highlighted a novel COX-2/CYP19A1 axis in the pathogenesis of MPM that can be pharmacologically targeted, consequently opening up new therapeutic options.

**Supplementary Information:**

The online version contains supplementary material available at 10.1186/s13046-021-02050-1.

## Background

Malignant mesothelioma (MM) is an aggressive tumor of the mesothelium, the thin tissue that lines the lung, chest wall, and abdomen. It is associated with exposure to asbestos, and has a latency period of 10–40 years between exposure and the onset of the disease [1–5]. Prognosis with MM is poor and median survival ranges from 8 to 14 months from diagnosis [6]. An estimate based on data from 2008 reported an average of 14,200 cases worldwide each year and where a peak incidence is expected to occur before 2030 [7, 8]. However, in some industrial countries where asbestos has not been banned its continuous and unregulated use will continue to affect global health even after its peak incidence has passed [9]. Asbestos, a ubiquitous environmental substances, is a complete carcinogen with tumor initiating and promoting activities. The risk of developing MM is related to the dimensions, heaviness and duration of exposure to asbestos fibers [10]. Following inhalation of thin asbestos fibers into the lung they penetrate into the pleural space and interact with mesothelial cells and inflammatory cells, initiating cyclical processes of tissue damage, repair and local inflammation. Mesothelial cells and macrophages release a variety of cytokines and growth factors that inhibit asbestos-induced cytotoxicity and induce inflammation and tumor promotion that ultimately lead to the onset of malignant pleural mesothelioma (MPM) [5]. Among the different mediators of inflammation, the cyclooxygenases (COXs) appear to be implicated in many solid tumors, including MPM [11, 12]. Cyclooxygenase-2 (COX-2) is an inflammatory cytokine-inducible enzyme that catalyzes the conversion of arachidonic acid into various structurally related prostaglandins (PGE), including PGE2, PGD2, PGF2a, PGI2 and thromboxane A2 [13, 14]. Interestingly, the synthesis of PGE2 mediated by COX-2 has a positive feedback on the expression of COX-2, which in turn leads to an increase in PGE-2 responsible of increasing the risk and development of malignant tumors [13, 14]. On this basis, it has been suggested that COX-2 inhibitors may be a good strategy for cancer prevention and therapy [15, 16]. COX-2 inhibitors have a cytotoxic effect in MPM cells and COX-2 expression contributes independently to other clinical and histopathological factors in determining short survival in MM patients [17–19]. Although the regulation of mRNA stability seems to be the most important regulatory step for COX-2 expression, several studies have reported that other mechanisms, such as transcriptional control or hypermethylation are involved in regulating COX-2 expression [20]. In cancer cells the altered posttranscriptional regulation of COX-2 is mediated by increased cytoplasmic mRNA binding of the mRNA stability factor human embryonic lethal abnormal vision-like protein HuR (HuR) [21]. In MPM cells, pro-inflammatory cytokines stimulation induces translocation of HuR from the nucleus to the cytoplasm and this contributes to COX‐2 mRNA stabilization and protein synthesis. In human MPM samples, the cytoplasmic expression of HuR is significantly correlated with high COX-2 expression and with poor survival [22]. COX-2 derived PGE-2 promotes tumor growth, enhancement of cellular proliferation, promotion of angiogenesis, stimulation of invasion/mobility, suppression of immune responses and inhibition of programmed cell death by binding to four types G protein-coupled receptors termed EP1, EP2, EP3, and EP4 (E-series prostanoid receptors ) [23, 24], that activate different signal transduction pathways. PGE2 is able to promote cancer cell growth through EP2 signaling that involves the activation of survival pathways, such as phosphoinositide 3-kinase (PI3K) and the protein kinase Akt and Extracellular Signal–Regulated Kinase 2 **(**ERK1/2), that have been implicated in asbestos-mediated mesothelial cell transformation [25–28]. PGE2-mediated activation of EP2 and EP4 receptors increase intracellular cyclic-adenosin monophosphate (cAMP) by activating adenylate cyclase [29]. cAMP stimulates expression of PKA-CREB-dependent genes including COX-2, aromatase (CYP19A1) [30]. CYP19A1 is the cytochrome P450 enzyme complex that converts C19 androgens to C18 estrogens. The biologically active estradiol (E2) exasperates pathologic processes including inflammation and influences the risk of cancer through an inflammation-related mechanism [31–33]. CYP19A1 has recently been identified in both MPM cells and specimens. CYP19A1 is expressed in 83 % of tissue samples from patients with MPM as a cytoplasmic protein and its expression is significantly associated with poor patient survival [34]. Furthermore, cytoplasmic expression of E2 was identified in MPM tissues with a trend towards a negative correlation between E2 levels and the median post-diagnosis survival time of patients [35]. Exemestane, a CYP19A1 inhibitor, decreases E2 level and induces death of MPM cells. In MPM xenografts, the daily exemestane therapy results in the reduction of the tumor mass and plasmatic E2 levels [35, 36]. These new findings, together with the known role of COX-2 in MPM, highlighted the possibility of a relationship between inflammation, COX-2 and CYP19A1 in malignant mesothelioma [37]. In this regard, data obtained from immunohistochemical (IHC) analysis of COX-2 and CYP19A1 on MPM samples encouraged further investigation into the role of COX-2 on CYP19A1 activation [22, 34]. It is plausible that PGE2 produced by the induction of COX-2 at the inflammation site after exposure to asbestos could increase CYP19A1 activity and, consequently, increase E2 levels which, in turn, contribute to asbestos carcinogenesis. However, the mechanism (s) by which COX 2 can contribute to mesothelioma pathogenesis remains to be addressed. For example, COXs ability to activate a variety of environmental carcinogens during early or late stages of carcinogenesis at extra-hepatic sites has been demonstrated. In the lung, COX-2 was involved in the conversion of tobacco procarcinogens into mutagens [38]. In the breast, PGEs stimulate aromatase activity and COX-2 inhibition has been shown to prevent estrogen-induced breast tumor formation [39, 40]. The aim of the present study is therefore, to understand the role of COX-2 and CYP19A1 and their possible cross-talk in MPM in order to develop possible strategies to prevent asbestos-induced carcinogenesis and create new therapeutic schemes for the targeted therapy of MPM.

## Materials and methods

### Cell lines

The human pleural MPM cell lines MSTO-211 H (MSTO) and NCIH-2452 (NCI) were obtained from the American Type Culture Collection (ATCC) (Rockville, Md) and Ist-Mes1, Ist-Mes2, and MPP89 were obtained from Genova Institute Culture Collection. Cell lines were cultured according to ATCC and Genova Institute Culture Collection protocols and gradually conditioned in Dulbecco’s Modified Eagle Medium/F12 + Glutamax (Thermo Fischer Scientific, Waltham, MA USA) supplemented with 10 % foetal bovine serum and antibiotics and maintained at 37 °C and 5 % CO2. To ensure that the cells are uncontaminated and correctly identified, cell lines were periodically tested for mycoplasma contamination by MycoFluor™ Mycoplasma Detection Kit (Thermo Fischer Waltham, MA USA) and cell morphology was monitored routinely and compared to cell morphology images, and the growth curve analysis was evaluated periodically.

### Cell treatments

Cell treatments were assessed in a monolayer culture condition by plating Ist Mes1, Ist Mes2 and MPP89 cells in T25 flask. After 24 h, 10nM PGE, or 35µM rofecoxib, a selective COX-2 inhibitor, or 35 µM exemestane, a CYP19A1 inhibitor, or 35µM rofecoxib with 35 µM exemestane was added for the time indicated in the experiments. Based on our previously published studies, we selected the concentration of 35 µM rofecoxib that induced a reduction in COX-2 expression and COX-2 mRNA levels in MPM cells (17) and the concentration of 35 µM exemestane that induced MPM cell death (35). Exemestane, rofecoxib and PGE2 were purchased from Sequoia Research Products, Pangbourne. United Kingdom. 1,5 MK2206 ( Selleck Chemicals Munich, Germany), an AKT inhibitor, was added to medium of Ist Mes2 cell 24 h before of treatment with 35µM rofecoxib or 35 µM exemestane, or 35µM rofecoxib and 35 µM exemestane combination for the time indicated in the experiments. At the end of treatment, the cells were then harvested and analyzed by reverse transcriptase-polymerase chain reaction (RT-PCR), western blot and fluorescence-activated cell sorting (FACS) as described below. The cytotoxic effect obtained with the exemestane and rofecoxib combinations was analyzed according to the Chou and Talalay method [41]. To determine the combination index (CI) Ist Mes1, Ist Mes2 and MPP89 cells were treated with 4 concentrations (1.4, 7, 35 and 175 µM) of exemestane or rofecoxib alone and with 35µM rofecoxib and 35 µM exemestane in combination. The trypan blue staining procedure was used for manual count of the number of live (unstained) and dead (blue) cells. All the treatments were done in triplicates. The inhibition of cell proliferation (fraction affected, Fa) was measured for all treatments and used to determine CI. CI values above 1.1 indicate antagonistic, 0.9 to 1.1 additive, 0.7 to 0.9 moderately synergistic, 0.3 to 0.7 synergistic, and < 0.3 strongly synergistic.

### Spheroid cell culture

Mesothelioma cells were grown to near confluency, dissociated into single cells with Accutase (Thermo Fischer Scientific, Waltham, MA USA) and plated in ultralow attachment plates (Corning® Ultra Low Attachment Corning, NY 14,831 USA). The plate was then centrifuged at 1000 rpm for 10 min to initiate cell-cell interaction and incubated for 48 h at 37 °C, 5 % CO2. Self-renewal potential of MPM spheroids (mesosphere) was tested by their disintegration to single-cell and replating in ultra-low attachment plates as described above. 35µM rofecoxib or 35 µM exemestane, or 35µM rofecoxib and 35 µM exemestane combination were added to MPM spheroids after manual disaggregation. Ninety-six hours later, the spheroids were photographed and measured with Scion Image Software and collected to analyzed by immunohistochemistry. Sphere-forming efficiency (%) was determined by dividing the number of spheres formed by the original number of seeded cells. The quotient was then multiplied by 100. All experiments were repeated in triplicate and media values were calculated.

### RNA isolation and RT-PCR assay of COX-2 and CYP19A1

Total RNA was prepared from cell culture using TRIzol Reagent (Thermo Fischer Scientific, Waltham, MA, USA) according to the manufacturer’s protocols and 4 µg were used for retro transcription (RT). cDNA was examined by quantitative polymerase chain reaction(PCR), conducted in the ABI PRISM 7000 sequence Detection System (Applied Biosystems, Foster City, CA). Quantitative PCR for the endogenous control glyceraldehyde-3-phosphate dehydrogenase (*GAPDH*) was carried out using a GADPH Assay on Demand (Applied Biosystems, Foster City, CA). Target sequences were amplified using the following primer pairs: GAPDH forward sequence 5*′-*GTCTCCTCTGACTTCAACAGCG-3*′ and* reverse sequence 5′-ACCACCCTGTTGCTGTAGCCAA-3*′;* CYP19A1 forward sequence: 5*′-*GACGCAGGATTTCCACAGAAGAG-3*′* and reverse sequence 5*′-*ATGGTGTCAGGAGCTGCGATCA-3*′*; COX-2 forward sequence 5*′-*CGGTGAAACTCTGGCTAGACAG-3*′* and reverse sequence 5*′-* GCAAACCGTAGATGCTCAGGGA-3*′* (OriGene Technologies, Inc. MD, USA). A standard curve for COX-2 or CYP19A1 was constructed using serial dilutions of a pool of cDNAs from MPM cells. Results were analyzed by using the Applied Biosystems analysis software and expression levels calculated from a linear regression of the standard curve. Results are given as COX-2 or CYP19A1 gene expression versus GAPDH expression to correct differences in the quantity of cDNA used in the PCR reaction. All quantitative PCR reactions for each sample were performed in triplicate.

### Protein extraction and western blot analysis

Briefly, 25–50 µg of proteins extracted by treating cells with ice-cold lysis buffer (20mM Tris pH 8, 1 % NP40, 10 % glycerol, 137 mM NaCl, 10 mM ethylenediaminetetraacetic acid, and inhibitor of protease and phosphatase) were separated by SDS-PAGE and transferred onto polyvinylidenedifluoride membrane. Membranes were blocked and blotted with relevant antibodies. Goat anti mouse or rabbit IgG horseradish peroxidase conjugated secondary antibodies (1:3,000) (Bio-Rad Laboratories; Hercules, CA, USA) were used. Antibody reaction was visualized by the chemiluminescence detection system (Clarity Western ECL Substrate Bio-Rad) and quantified using Scion Image program. Proteins were probed with antibodies against CYP19A1, COX-2, p27, p21, p53, cyclin D1 and caspase-3 (Santa Cruz Biotechnology, Santa Cruz, CA, USA), cleaved PARP (Cell Signaling Technology, Danvers, MA, USA ) and actin or vinculin (Sigma, Saint Louis Missouri, USA). Actin or vinculin or tubulin were used as a loading control. For statistical analysis protein band intensities were normalized to actin or vinculin protein (relative band intensity), and to untreated samples (CNTR). The experiments were performed in triplicate.

### Flow cytometry

Cell cycle analysis was performed by flow cytometry. Cells were fixed in 70 % ethanol and stored at -20 °C overnight. Fixed cells were treated with 1 mg/ml RNase A (Thermo Fischer Scientific, Waltham, MA, USA) for 1 h at 37 °C and DNA was stained with Propidium Iodide (Sigma St. Louis, MO, USA). Samples were acquired with a Guava EasyCyte 8HT flow cytometer (Merck Millipore Billerica, Massachusetts,USA). Cell cycle distribution was shown.

### Immunohistochemical analysis of mesopheres and tissues samples

After 5 days in culture, mesospheres were pelleted and fixed in buffered formalin for 24 h before being processed for paraffin embedding. 2 μm sections were cut for IHC analysis. MM samples were collected at the time of the initial biopsy to establish the diagnosis of MM in the Mesothelioma Biobank of the Coorporate National health public hospital SS Antonio e Biagio and C. Arrigo in Alessandria. Histological specimens were obtained from formalin fixed paraffin embedded (FFPE) tissue; 3–5 μm thick tissue slides were cut using a sledge microtome and mounted onto pre-coated adhesive glass slides. IHC analysis was performed using the following antibodies: 17-beta-estradiol (rabbit polyclonal antibody; Biogenex) after antigen retrieval in citrate buffer (pH6 for 20 min), Cox-2 (H-3) (mouse monoclonal antibody, 1:250) after antigen retrieval in citrate buffer (pH6 for 20 min). IHC staining was performed in an automated autostainer (BOND-III, Leica, Milan, Italy) by a biotin-free polymeric horseradish peroxidase (HRP)-linker antibody conjugate system (Bond polymer refine, Leica). The IHC staining of the samples was evaluated by immunoreactivity score (IRS); this evaluation system includes staining intensity and quantitative count of positive cells. Briefly, staining intensity was graded as: 0, negative; 1, weak; 2, moderate or 3 strong and the percentage of positively stained cells was scored as: 0, negative; 1, ≤ 10 %; 2, ≥ 10 ≤ 50 %; 3, > 50-≤80 % or 4, > 80 %. These two scores were multiplied to calculate the IRS, which ranged from 0 to 12 as follows: 0–1, negative, 2–3, mild expression, 4–8, moderate expression or 4–12, strongly expression.

### Statistical analysis

All experiments were performed in triplicate. Data are presented as mean values ± SD of at least three experiments performed in triplicate. Comparing treatment outcomes were tested for statistical differences using the Student t-test for paired data. Statistical significance was assumed at a *P*-value of ≤ 0.05. The statistical significance of the association between proportions of categorical data was assessed by Fisher’s exact test.

## Results

### Characterization of COX-2 and CYP19A1 in MPM

**Fig. 1:**
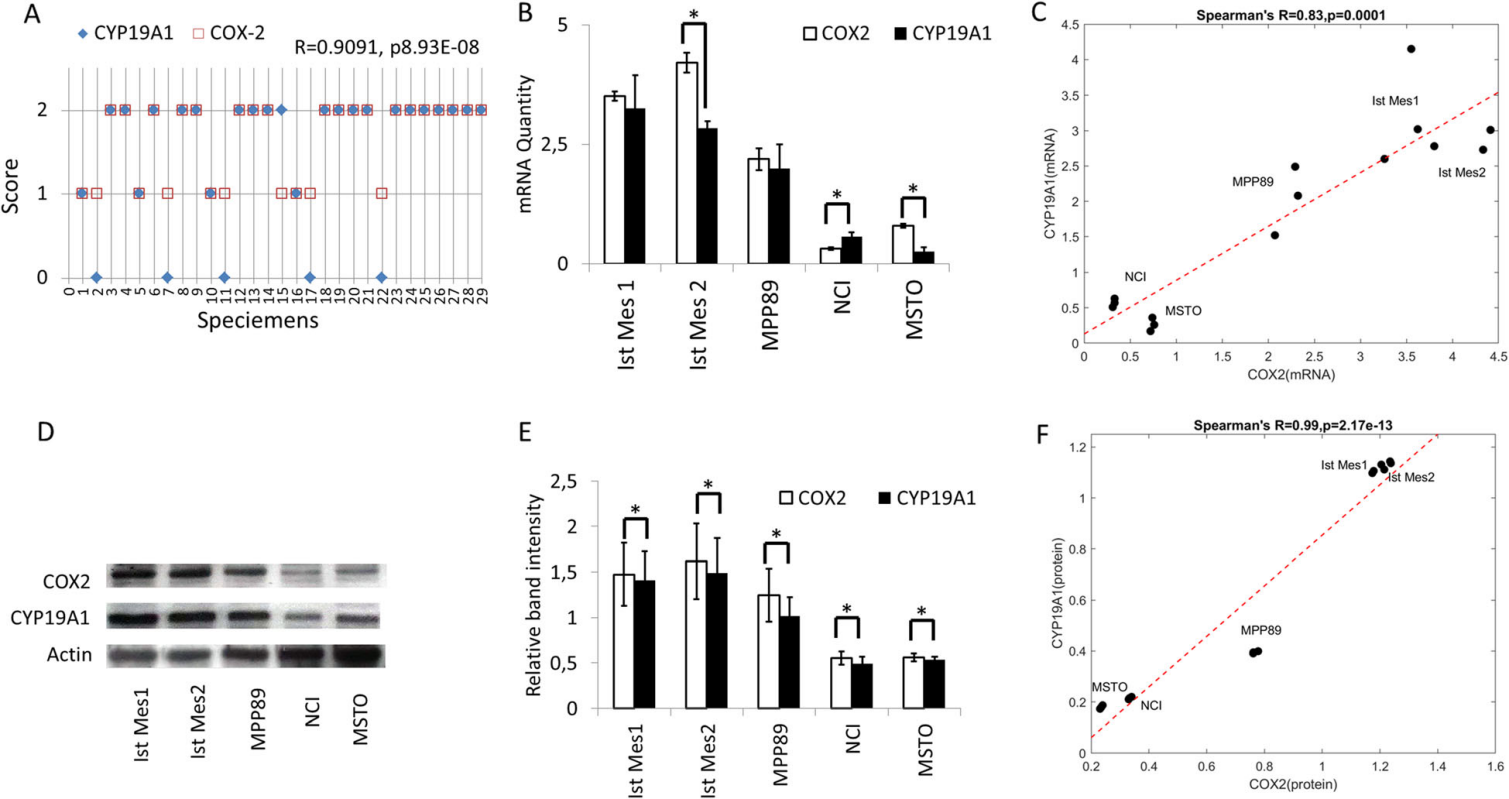
Characterization of COX-2 and CYP19A1 in MPM tissues and cell lines. **A** The graph represents the correlation between COX-2 and CYP19A1 staining in 29 MPM specimens. The score indicates the intensity of COX-2 and CYP19A1 staining: 0, absent, 1, low; 2, high. **B** The graph represents the means ± SD of three independent quantifications by RT-PCR of COX-2 and CYP19A1 mRNA levels in Ist Mes1, Ist Mes2, MPP89, NCIH-2452 (NCI), and MSTO-211 H (MSTO) cells. **C** Graphical representation of Spearman’s correlation between COX-2 and CYP19A1 mRNA levels in MPM cells **D** Representative experiment out of 3 independent western blots on COX-2 and CYP19A1 expression in MPM cell lines; **E** The graph represents the average of 3 experiments for the detection of COX-2 and CYP19A1 expression in the five MPM cell lines by western blot. Protein expression was measured by Scion Image Software as the intensity of each band relative to the loading control (relative band intensity). **F** Graphical representation of Spearman’s correlation between COX-2 and CYP19A1 protein expression in MPM cells.*, statistically significant effects (paired Student t test *P* < 0.05) compared to CNTR

We reported in previous studies the staining of COX-2 and CYP19A1, respectively in the 29 specimens of MPM (Suppl. Table 1) [22, 34]. Reprocessing of old data using Spearman’s rank correlation coefficient test showed that COX-2 was positively correlated with CYP19A1 protein expression (*r* = 0.9091, p8.93E-08) (Fig. 1a) encouraging further investigation on MPM cells. The quantization of COX-2 and CYP19A1 mRNA in five mesothelioma cell lines (MPP89, Ist Mes2, Ist Mes1, MSTO and NCI) by RT-PCR revealed that Ist Mes-1, Ist Mes-2, and MPP89 cell lines endowed higher mRNA COX-2 levels and exhibited higher mRNA CYP19A1 levels. In contrast, MSTO and NCI cells, characterized by lower mRNA COX-2 levels, showed lower mRNA CYP19A1 levels (Fig. 1b). The Spearman’s correlation coefficient used to measure the strength of the relationship between COX-2 and CYP19A1 mRNA determined their positive correlation in MPM cell lines, (*r* = 0.83, *p* = 0.0001; Fig. 1c). COX-2 and CYP19A1 expression was also detected in MPM cell lines by western blot (Fig. 1d). COX-2 and CYP19A1 were more expressed in Ist Mes-1, Ist Mes-2, and MPP89 cell lines compared to NCI and MSTO cells. Relative band intensity quantification showed different levels of COX-2 and CYP19A1 in five MPM cell lines (Fig. 1e). COX-2 was positively correlated with CYP19A1 protein expression in MPM cell lines by Spearman’s correlation coefficient, (*r* = 0. 99, *p* = 2,17e-13; Fig. 1f). Based on the research findings described above and these results, one can see that the upregulation of COX-2 expression is closely correlated to the expression of CYP19A1 in MPM cell lines and tissues samples.

### CYP19A1 modulation by COX-2 occurred at transcriptional level

**Fig. 2:**
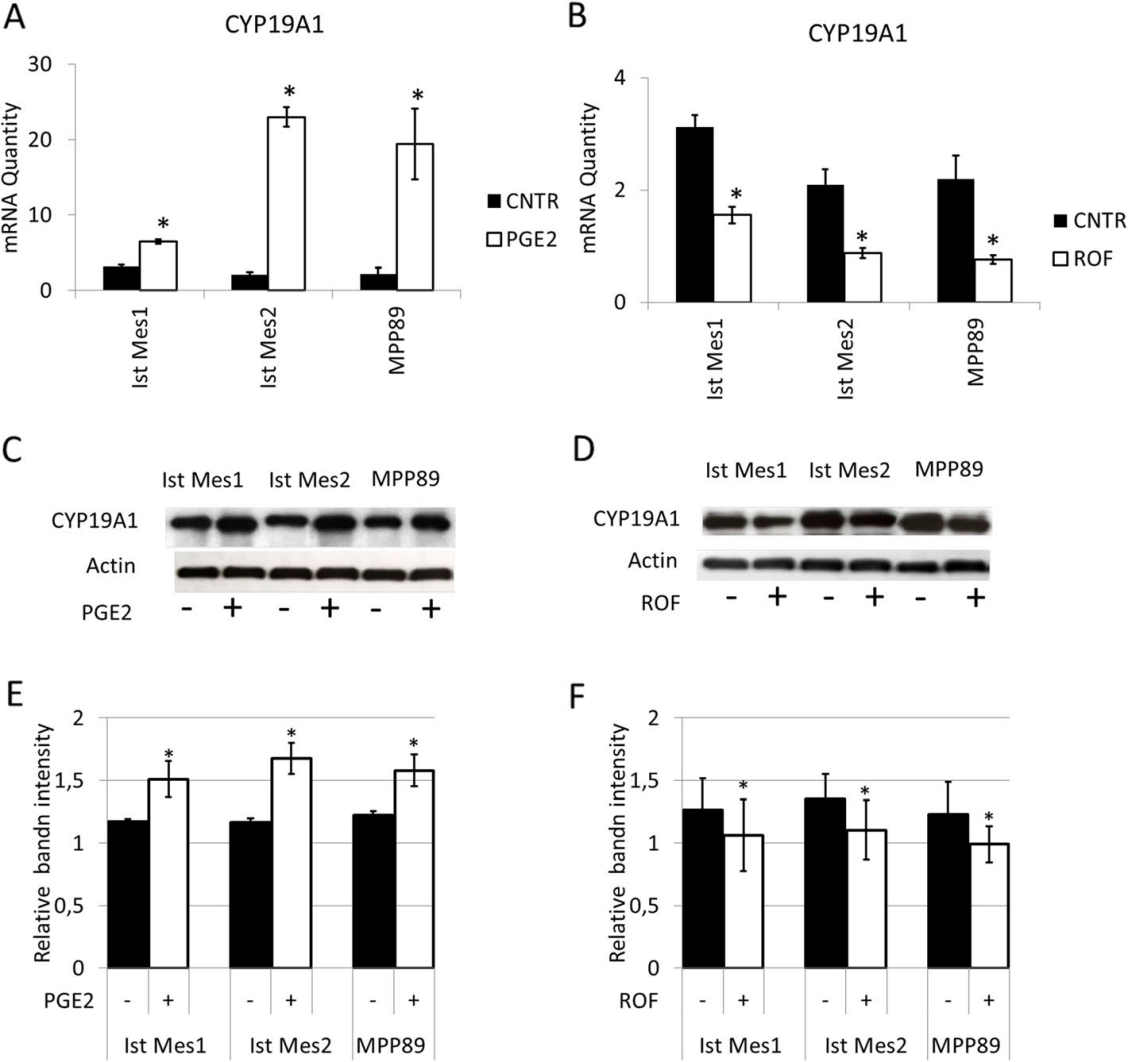
Modulation of CYP19A1 by COX-2. The graphs represent the means ± SD of three independent quantifications by RT-PCR of CYP19A1 mRNA levels in Ist Mes1, Ist Mes2 and MPP89 cells 3 h after adding PGE2 (**A**) or 35µM rofecoxib (ROF) versus untreated cells (CNTR) (**B**). Representative western blots analysis of CYP19A1 expression in Ist Mes1, Ist Mes2 and MPP89 cells 24 h after adding PGE2 (**C**) or rofecoxib (**D**). Graphs (**E**, **F**) represent the mean ± SD of three independent quantifications of protein band intensities normalized to the loading control from a western blot analysis.*, statistically significant correlation between COX-2 and CYP19A1 (paired Student t test *P* < 0.05)

To verify the hypothesis that COX-2 can upregulate CYP19A1, MPM cells with a higher baseline CYP19A1 level (MPP89, Ist Mes1 and Ist Mes2) were treated with 10nM PGE-2 and analyzed for CYP19A1 expression. Real time PCR analysis showed a significant increase in CYP19A1 mRNA levels after three hours from the addition of PGE-2 to MPM cells when compared to untreated cells (CNTR) (Fig. 2a). We had previously shown that Ist-Mes-1, Ist-Mes-2 and MPP89 cell lines incubated with 35 µM rofecoxib, the COX-2 inhibitor, disclosed a significant decrease in COX-2 expression, at protein and mRNA levels [17]. In addition, treatment with rofecoxib in MPM cells dropped the levels of PGE-2 compared to controls. Accordingly, we used 35µM rofecoxib to investigate the effect of COX-2 inhibition on the modulation of CYP19A1. As shown by RT-PCR analysis, the addition to MPM cells of 35µM rofecoxib resulted in a decrease in CYP19A1 mRNA expression when compared to respective untreated MPM cells (Fig. 2b). By western blot, the protein expression of CYP19A1 in MPP89, Ist Mes1, and Ist Mes2 cells, after 24 h of treatment with 10nM PGE2 (Fig. 2c, e) or rofecoxib (Fig. 2d, f) was decreased or increased, respectively. These results indicate that inhibition of COX-2 by rofecoxib reduces the expression of CYP19A1, suggesting that the expression of CYP19A1 was modulated by PGE2 at the transcriptional level in MPM cells.

### Inhibition of COX-2 enhances the effect of the CYP19A1 inhibitor on cell proliferation of MPM

**Fig. 3:**
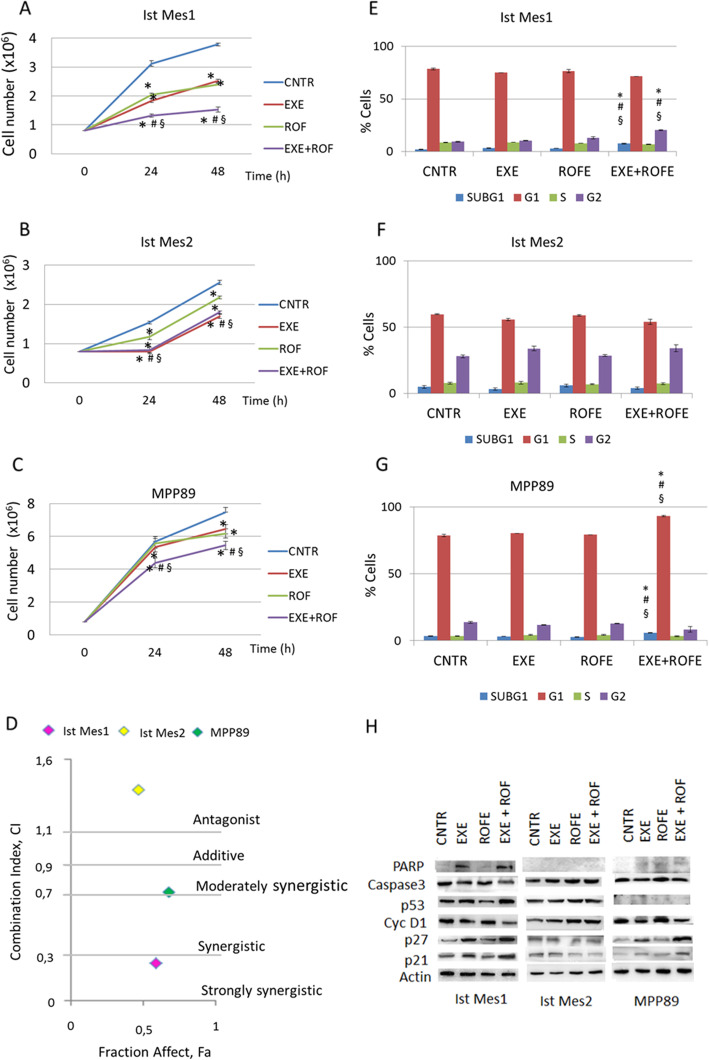
Effects of exemestane, rofecoxib and exemestane and rofecoxib combination on cell growth in Ist Mes1, Ist Mes2 and MPP89 cells. MPM cells were cultured in the absence (CNTR) or presence of 35µM exemestane (EXE) or 35µM rofecoxib (ROF) or 35µM exemestane and 35µM rofecoxib combination (EXE + ROF) for 24 and 48 h. The graphs represent the number of vital Ist Mes1 (**A**), Ist Mes2 (**B**) and MPP89 (**C**) cells. **D** The combination index (CI)-plot of MPM cells treated with EXE + ROF. CI values above 1.1 indicate antagonistic, 0.9 to 1.1 additive, 0.7 to 0.9 moderately synergistic, 0.3 to 0.7 synergistic, and < 0.3 strongly synergistic. Cell cycle analysis after propidium iodide staining was performed by flow cytometry in MPM cells untreated (CNTRL) or treated with EXE, ROF and EXE + ROF for 48 h. The percentages of Ist Mes1 (**E**), Ist Mes2 (**F**) and MPP89 (**G**) cells in different phases of cell cycle were reported in graphs. Data are expressed as mean ± SD of at least three independent experiments. Statistically significant effects (paired Student t test *P* < 0.05) compared to CNTR *, EXE # or ROF § **H** Western blots of cell cycle and apoptosis protein expression in Ist Mes1, Ist Mes2 and MPP89 cells treated with EXE, ROF and EXE + ROF for 48 h

COX-2 and CYP19A1 inhibitors were used to identify pathways involved in modulating the expression of CYP19A1 by COX-2 in MPM cells. In this regard, 35µM rofecoxib certainly downregulated COX-2 and 35µM exemestane, causing modest cell death, were used. First, the effect of 35µM of rofecoxib or 35µM of the aromatase CYP19A1 inhibitor exemestane or their combination on the cell proliferation of Ist Mes1, Ist Mes2 and MPP89 cell lines after 24 and 48 h of treatment was evaluated by analyzing cell vitality (Fig. 3a,b,c). The Ist Mes1 (Fig. 3a) and Ist Mes2 (Fig. 3b) cell lines were found to be more sensitive to 48 h exemestane treatment with approximately 60 % live cells compared to 100 % of untreated cells. Treatment with rofecoxib for 48 h leaves 60 %, 85 and 88 % of live cells in Ist Mes1 (Fig. 3a), Ist Mes2 (Fig. 3b) and MPP89 cells (Fig. 3c), respectively, compared to 100 % of untreated cells. Combination of exemestane with rofecoxib for 48 h further reduces the number of live cells in Ist Mes1 (44 % of live cells versus untreated) (Fig. 3a) and MPP89 cells (71 % of live cells versus untreated) (Fig. 3c) but not in the Ist Mes2 cell line (73 % of live cells versus untreated) (Fig. 3b). The calculation of the combination index (CI) at 48 h indicates a strongly synergistic effect in Ist Mes1 cells, moderately synergistic effect in MPP89 cells and antagonist effect in Ist Mes2 cells (Fig. 3d). The analysis by flow cytometry of the cell cycle of Ist Mes2 cells treated for 48 h in monotherapy and in combination did not show any perturbation of cell cycle compared to untreated cells (Fig. 3f). The cell cycle analysis of the other two MPM cell lines treated with the drug combination showed a significant increase in the percentage of the cells, although modest, in subG1 and G2 in Ist Mes1 cells (Fig. 3e) and in subG1 and G1 in MPP89 cells (Fig. 3 g) compared to the respective single treatment and untreated cells. Expression of caspase-3, cleavage of poly(ADPribose) polymerase (PARP) (the substrate of caspase-3, an early index of apoptosis) and cell cycle modulator proteins (p53, p21, p27 and Cyclin D1) were examined by western blot in MPM lines after 48 h of rofecoxib and exemestane combination treatment or alone (Fig. 3 h). The quantification of protein band intensities are shown in Supplementary Fig. 1. Weakly modulation of apoptotic and cell cycle regulatory proteins were detected in Ist Mes1, and MPP89 cells after exemestane, but not upon rofecoxib treatment. Combination of the two drugs in Ist Mes1 and MPP89 cells resulted in increased cleaved-PARP, reduction of caspase3, downregulation of cyclin D1 and upregulation of p53 (only in Ist Mes1 cells), p21 and p27 protein expression confirming apoptosis and perturbation of the cell cycle, in line with the results highlighted by flow cytometric analysis. These results suggested that COX-2 inhibition potentiates the antiproliferative activity of CYP19A1 inhibitor in MPM cells, characterized by overexpression of COX-2 and CYP19A1.

### AKT activation is involved in the synergistic effect of exemestane and rofecoxib combination

**Fig. 4:**
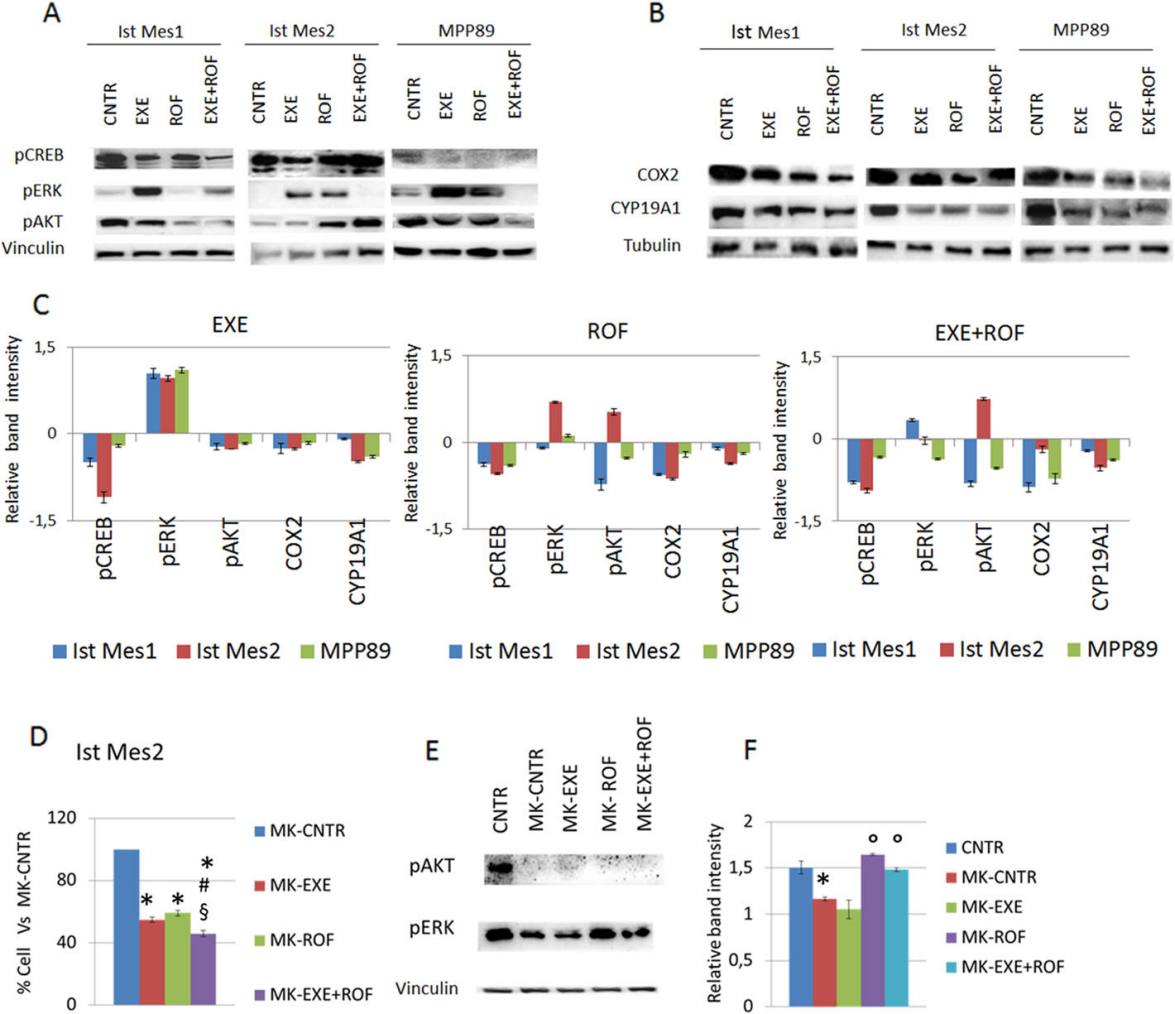
AKT and ERK phosphorylation are implicated in the combined action of rofecoxib and exemestane. **A** Representative experiment out of three independent western blot analyses of pCREB, pERK and pAKT expression in Ist Mes1, Ist Mes2 and MPP89 cells treated with 35µM exemestane (EXE) or 35µM rofecoxib (ROF) or 35µM exemestane and 35µM rofecoxib combination (EXE + ROF) for 30 min. **B** Representative western blot analyses of COX-2 and CYP19A1 expression in Ist Mes1, Ist Mes2 and MPP89 cells treated with 35µM exemestane (EXE) or 35µM rofecoxib (ROF) or 35µM exemestane and 35µM rofecoxib combination (EXE + ROF) for 24 h. **C** The graphs represent the mean ± SD of three independent quantifications of protein band intensities normalized to the loading control and then in comparison to the untreated sample (relative band intensity). **D** The graph represents the mean ± SD of three independent cell survival rates after pre-incubation with MK-2206 and subsequently treated with exemestane (MK-EXE), or rofecoxib (MK + ROF) or exemestane and rofecoxib combination (MK-EXE + ROF) compared to untreated (100 % of cell alive). **E** Representative western blot analyses of pAKT and pERK expression in Ist Mes2 cells pre-incubated with MK-2206 and after treated with exemestane (MK-EXE), or rofecoxib (MK + ROF) or exemestane and rofecoxib combination (MK-EXE + ROF). **F** The graph represents the mean ± SD of three independent quantifications of protein band intensities normalized to the loading control. Statistically significant effects (paired Student t test *P* < 0.05) compared to CNTR *, EXE # or ROF § or MK-CNTR °

MPM cells treated with rofecoxib or exemestane alone or in association were analyzed for the protein expression of pCREB, pAKT and pERK after 30 min (Fig. 4a) and CYP19A1 and COX-2 (Fig. 4b) after 48 h. The band intensity of each protein from the lysate of cells, treated with combination of exemestane or rofecoxib or in monotherapy, was quantified (Fig. 4c). After 48 h of treatment of MPM cells with drugs alone or in combination, a reduction of COX-2 and CYP19A1 expression was detected in Ist Mes1, Ist Mes2 and MPP89 cells by western blot (Fig. 4b, c). We analyzed pCREB, pAKT and pERK expression in MPM cells after treatment for 30 min with exemestane or rofecoxib alone or in combination. Exemestane treatment induced an increase in pERK and a reduction of pAKT and pCREB expression in all cell lines (Fig. 4a, c). Rofecoxib acted differently on the expression of protein analyzed in MPM cells. The Ist Mes1 cell lines (Fig. 3a), more sensitive to rofecoxib (60 % of live cells versus untreated) showed a reduction in pCREB, pAKT and pERK, the Ist Mes2 cells (Fig. 3b) less sensitive to rofecoxib (85 % of live cells versus untreated) showed a reduction of pCREB and an increase in the expression of pERK and pAKT, the MPP89 cells (Fig. 3c), also less sensitive to rofecoxib (88 % of live cells versus untreated) showed a reduction of pCREB and pAKT and an increase in pERK (Fig. 4a, c). Combination treatment with rofecoxib and exemestane lowered pCREB expression in all cell lines, pAKT in Ist Mes1 cells (44 % of live cells versus untreated, Fig. 3a) and MPP89 cells (71 % of live cells versus untreated, Fig. 3c) while pERK was up- and down- modulated in Ist Mes1 and MPP89 cells, respectively (Fig. 4a, c). In Ist Mes2 cells (73 % of live cells versus untreated, Fig. 3b) the drug combination induced an increase in pAKT and no modulation of ERK activation (Fig. 4a, c). Overall, it appears that pAKT, pERK were involved in the response of an individual drug or to drug combination. The comparison of protein modulations with respective synergistic effects of the drug combination suggests that while ERK activation could be responsible for a strong synergism, the downregulation of pAKT was pivotal for the synergistic effect shown in Ist Mes1 and MPP89 cells (Fig. 3d). To further explore the molecular mechanism by which AKT activation interfers with the efficacy of combination treatment, we tested MK2206, a pAKT inhibitor, on Ist Mes2 cells. Treatment with 1.5 µM MK2206 of the Ist Mes2 cell line led to 86 % live cells compared to untreated cells. Ist Mes2 cell treatment for 24 h with exemestane and rofecoxib alone or in combination after pre-incubation with MK2206 resulted in 55 %, 59 %, and 46 % of live cells, respectively (Fig. 4d), while the same treatment without MK2206 pre-incubation displayed a cell life of 66 % for exemestane, 85 % for rofecoxib and 62 % for the combination of exemestane and rofecoxib (Fig. 3b), suggesting that the absence of pAKT improves the response to treatment especially with combination treatment. The analysis of protein expression from Ist Mes2 cell lysate after the different treatments, in addition to confirm the absence of pAKT expression in the samples pre-incubated with MK2206, revealed an upregulation of pERK expression upon treatment with AKT inhibitor in mono and combination treatments (Fig. 4e, f). Interestingly, the combination therapy with MK2206 upregulates pERK in Ist Mes2 cells, as well as observed in Ist Mes1 cell that showed a synergistic effect of exemestane and rofecoxib, suggesting that the inhibition of AKT promotes an activation of ERK pathway. Here, we demonstrated that the inhibition of AKT activation, is involved in the synergistic effect of exemestane and rofecoxib combination.

### COX-2 modulates the CYP19A1 activity as E2 production

**Fig. 5:**
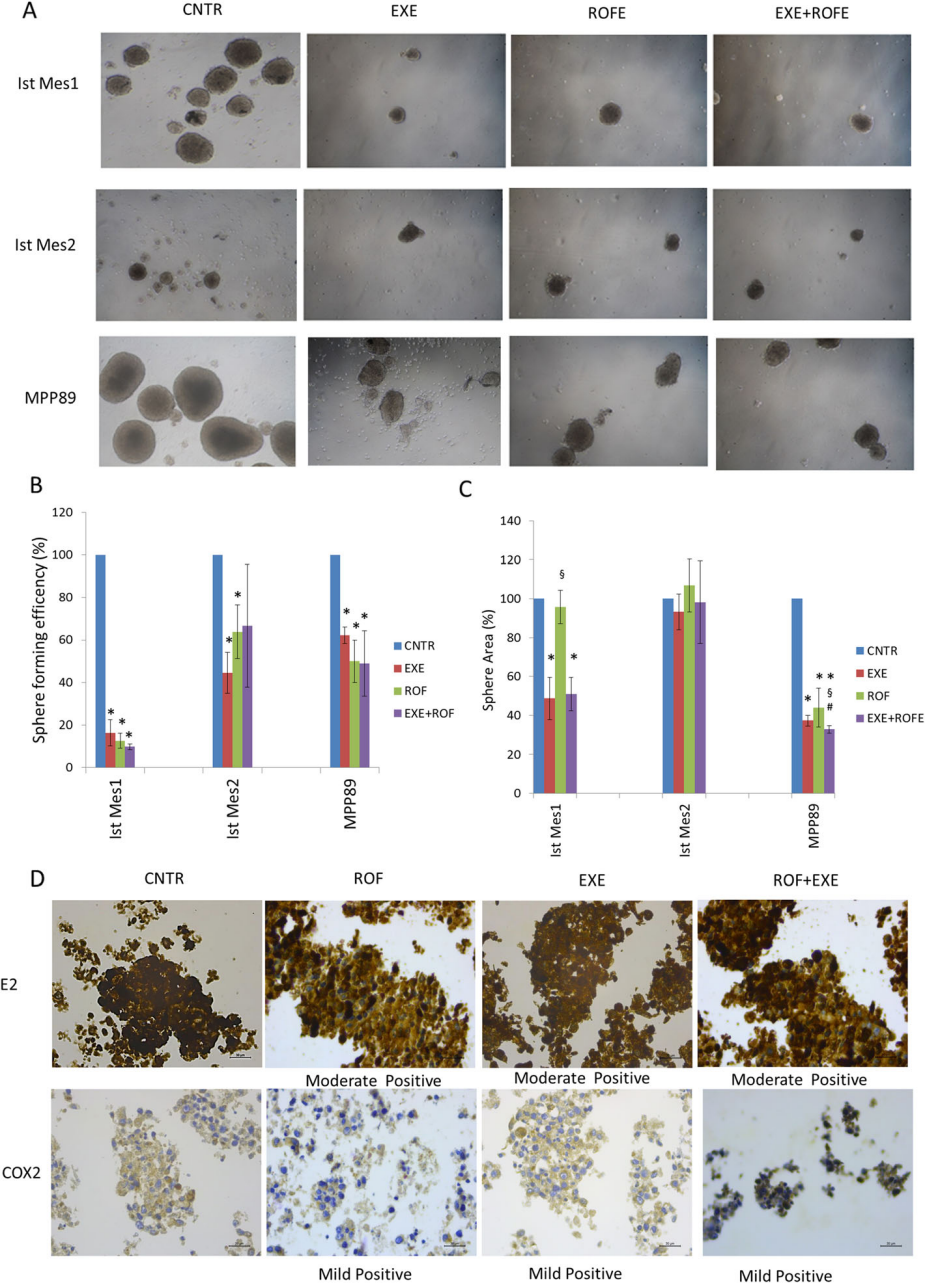
Effect of COX-2 and CYP19A1 inhibitor on the mesospheres formation. Spheres MPM were treated with exemestane (EXE) or rofecoxib (ROF) or exemestane and rofecoxib combination (EXE + ROF) Ninety-six hours later, the spheroids were photographed (**A**) and sphere-forming efficiency (**B**) and size (**C**) were determined. All experiments were repeated in triplicate and media values were calculated. Statistically significant effects (paired Student t test *P* < 0.05) compared to CNTR *, EXE # or ROF §. **e** IHC staining of COX-2 and E2 in MPP cells after treatment

To explore the efficacy of COX-2 and CYP19A1 inhibitors in proxy preclinical models of MPM cells, we analyze the effect of combination treatment of COX-2 and CYP19A1 inhibitors in mesosphere formation. Ist Mes1, Ist Mes2 and MPP89 cell lines were capable of forming mesospheres after COX-2 or CYP19A1 inhibitor treatment alone or in association (Fig. 5a). Mesospheres in control and treatment group showed difference in size and sphere forming efficiency. Sphere forming efficiency upon treatments were smaller than in the control group, especially in Ist Mes1 cells (Fig. 5b). The quantification of sphere sizes, expressed as sphere area, confirmed the reduction of sphere size in Ist Mes1 and MPP89 cells after treatment (Fig. 5c). These data indicate that COX-2 or CYP19A1 inhibitor alone and in association exert a marked reduction in sphere-forming efficiency and size especially in Ist Mes1 and MPP89 cell lines. The possible involvement of COX-2 on the CYP19A1 activity such as E2 production, was investigated by the IHC analysis. Due to the reduced size and number of spheres obtained in Ist Mes1 and Ist Mes2 cells after drug treatment, they failed to produce sufficient material for the IHC analysis that was possible only in MPP89 spheres (Fig. 5d). The immune reactive score (IRS) indicates strong staining for E2 and moderate for COX-2 in untreated MPP89 spheres. After treatment, a reduction of COX-2 and E2 staining was observed independently from monotherapy or in association treatment (Fig. 5d). These data indicate that COX-2 and E2 are involved in reducing the size and sphere-forming efficiency in MPP89 spheres. Furthermore, the COX-2 inhibitor acts on the CYP19A1 activity and the CYP19A1 inhibitor acts on the modulation of COX-2, suggesting an interplay between E2 and COX-2.

### COX-2 and E2 expression in MPM specimens

**Fig. 6:**
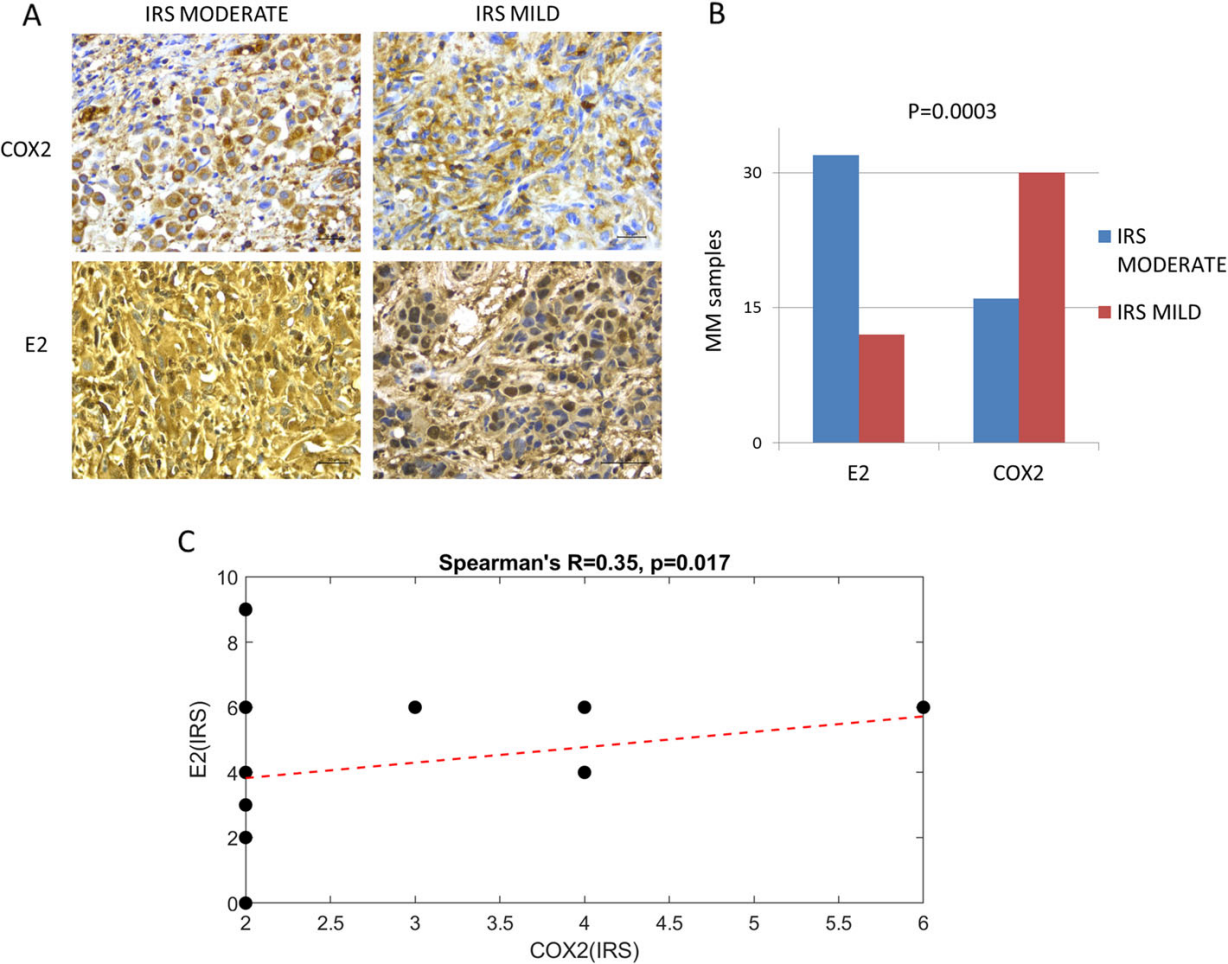
COX-2 and E2 expression by IHC in archival malignant pleural mesothelioma tumor specimens. **A**, Representative IHC of COX-2 and E2 in MPM and their relative immunoreactivity score (IRS). The scale bar is 30 μm. **B**, Graph shows the number of MPM samples with E2 and COX-2 moderate and mild IRS by IHC. **C**, graphical representation of Spearman’s correlation between COX-2 and CYP19A1 IRS value in MPM specimens

IHC staining for COX-2 and E2 were analyzed in paraffin-embedded tumor tissue samples from 46 MPM patients, 31 male and 15 female ranging between the age of 45 to 80 and 3 normal control subjects. We found cytoplasmatic staining for E2 and COX-2 in all normal pleura and in 46 mesothelioma samples with different percentage of positively stained cells between the tumor specimens (Fig. 6A). The graph of IRS in Fig. 6B indicates moderate and mild E2 staining in 33 and 12 samples, respectively, and moderate and mild COX-2 staining in 16 and 30 samples, respectively, of the 45 tumor tissues showing a significant difference in proportion between moderate and mild IRS between E2 ( mainly moderate) and COX-2 ( mainly mild). 17 (38 %) of the 45 samples displayed moderate IRS for E2 and mild IRS for COX-2 (moderate-mild), 28 (62 %) of the 45 samples showed the same IRS for E2 and COX-2, of these 16 were moderate-moderate and 12 were mild-mild. COX-2 and E2 association was assayed by Fisher’s exact test *P* = 0.0003. Furthermore, the correlation between COX-2 and E2 in the MPM samples was confirmed by using the IRS value to calculate the Spearman correlation coefficient (*R* = 0.35, *p* = 0.017; Fig. 6C).

## Discussion

The mechanism underlyng the pathogenesis of MPM remain unclear. Based on our previous research findings showing that COX-2 and PGE2 contribuite to the tumorigenicity of MPM, this study highlights the stimulating action exerted by COX-2 on the expression of CYP19A1 in MPM in order to better understand the pathways linking chronic inflammation associated with oncogenic transformation, consequently opening new preventive and therapeutic strategies for MPM. One previous report discussed the possible relationship between the inflammation involved in the progression of MPM, COX-2 and CYP19A1 suggesting a possible positive correlation [37]. This idea was later supported by comparing IHC analysis for COX-2 and CYP19A1, performed on the same MPM samples in different studies, where a significant positive correlation between the expression of COX-2 and CYP19A1 was observed [22, 34]. Based on this, the expression and mRNA levels of COX-2 and CYP19A1 in five MPM lines were assayed showing a positive correlation between COX-2 and CYP19A1 in the cells and in the MPM patient specimens. Further investigation was carried out on MPM lines by adding PGE2 or inhibiting it with rofecoxib, showing higher and lower presence of CYP19A1 in MPM cells, respectively, once again supporting the relationship between COX-2 and CYP19A1 that through a positive feedback loop sustains cell proliferation in MPM. On the other hand, E2 produced by CYP19A1 promotes MPM cell growth [35, 36]. Thus reducing CYP19A1 expression via COX-2 and inhibiting CYP19A1 activity with an CYP19A1 inhibitor may be an effective strategy to block the interplay COX-2 - CYP19A1. Notable, combination aromatase inhibitor plus COX-2 inhibitor therapy has shown a synergistic antitumor effect in several preclinical studies, including lung cancer, and was evaluated in several clinical trials [42–44]. These data, together with our previous reports, showing the action of exemestane on MPM cells, provided support for the use of combined COX-2 and CYP19A1 inhibitors to influence the cellular proliferation and identify possible pathways involved in the COX-2 and CYP19A1 interaction [34–36]. Thus, the combination of COX-2 and CYP19A1 inhibition, on cell proliferation assessed by the CI, was strongly synergistic in Ist Mes1 cells, moderately synergistic in MPP89 cells and antagonist in Ist Mes2 cell line. This result was useful for identifying the key pathways involved in the COX-2 and CYP19A1 interaction. Cell cycle analysis of the cell lines exhibiting a synergistic effect after treatment with the combination of COX-2 and CYP19A1 inhibitors showed, albeit modest, a significant increase in subG1 compared to single inhibitors suggesting an increase in apoptotic cells. Apoptosis was confirmed by caspase-3 activation and PARP cleavage in Ist Mes1 and MPP89 cells after combination of rofecoxib and exemestane. Caspase-3 is known to be one of the key executioners of apoptosis because its activation causes the cleavage of downstream important substrates, like PARP, which is the hallmark of caspase-dependent apoptosis. The balance between cellular levels of Cdk inhibitors, including p27Kip1 and p21Waf1/Cip1, regulates cell cycle progression [45]. p27Kip1 is a potential Cdk inhibitory tumor suppressor gene and p21Waf1 functions as an apoptosis-promoting protein [45, 46]. In addition, the regulation of p21 is dependent and independent on the presence of functional p53, a transcriptional regulator that mediates cell cycle arrest following DNA damage [46, 47]. In our cell model, MPP89 cells did not express p53, while Ist Mes1 and Ist Mes2 cells endowed p53, whose expression in the Ist Mes1 cell line was increased after treatment with exemestane or exemestane and rofecoxib combination. In Ist Mes1 and MPP89 cells the expression levels of p27Kip1, p21Cip1 were increased while the levels of cyclin D1, which plays a central role in the progression of cell cycle, were inhibited by the combined treatment of rofecoxib and exemestane. p21 can block cell cycle progression and keep cells in either G1 [48] or G2 phase [49] and a close relationship between p21 and drug-induced apoptosis is well known in MPM cell lines [17, 35, 50, 51]. We can hypothesize that p21 could also play a fundamental role in the induction of apoptosis by the combination of exemestane with rofecoxib. In our previous report, we indicate an essential role of reactive oxygen species (ROS) in the antiproliferative effect of exemestane in MPM cells, including Ist Me1 and MPP89 cells. In this regard, other studies reported that the action of rofecoxib is associated with an increase of ROS in different cell types [52, 53]. Oxidative stress is known to induce p21 expression through a mechanism that is independent of p53 [54] and this could take place in MPP89 cells. On the other hand, under stress, p53 induces transcriptional targets, such as p21, which lead to cell death by apoptosis [55]. This could occur in Ist Mes 1 cells which, unlike the MPP89 cell line, express p53. In light of these results, this study explored the regulatory pathway involved in the induction of apoptosis in MPM. Akt kinase, a serine/threonine kinase of the PI3K/Akt signaling pathway is important for tumor cell survival. The PI3K-AKT pathway is frequently activated and drives human cancer, making it an excellent candidate for therapeutic intervention [56]. AKT is frequently activated in MPM cells and elevated levels of Akt activity were found in 65 % of human mesothelioma specimens thus, the PI3K-AKT signaling pathway is a potential therapeutic target for MPM [57–59]. It is worthy to note that exemestane inhibits proliferation and induces apoptosis in MPM cells through modulation of the Akt/CREB signaling pathway [34, 35]. In addition exemestane acts in MPM cells through the generation of ROS, up-regulation of p-ERK and down-regulation of p-STAT [52]. Bearing this in mind, we thought that AKT and ERK activation could also be targets of rofecoxib in cells that showed a synergistic effect and therefore we investigated the modulation of ERK and AKT activation after treatment with the single inhibitors of COX-2 and CYP19A1 or in combination. Rofecoxib was shown to act on cell proliferation of the most sensitive Ist Mes1 cells by targeting AKT and ERK activation. The synergistic effect of the rofecoxib and exemestane combination resulted in the down-activation of AKT unveling a key role of AKT activation in the combined action of COX-2 and CYP19A1 inhibition. pAkt involvement was detected in all three MPM cell lines treated with combined exemestane and rofecoxib, despite each of them showed different response to the treatment. At this regard, pAKT was down regulated in the cells showing a synergistic effect to the drug combination and was up-regulated in the Ist Mes2 cell line showing an antagonistic effect. Thus, the pretreatment of Ist Mes2 cells with the pAKT inhibitor (MK2206) reverted the antagonistic effect of the combination COX-2 and CYP19A1 inhibitors by down-activation of AKT and up-activation of ERK. In this perspective, we can envision the activated form of AKT as a potential predicting factor of response to the combined regimen. It is well known that ERK1/2 can have pro-survival and pro-apoptotic functions in cells depending on the context and stimulus. In MPM cells, ERK activation after treatment with exemestane induced cell death by ROS production [52, 60, 61]. Following oxidative damage, ERK1 / 2-mediated apoptosis occurs through activation of caspase-3 and inactivation of the PI3K / Akt pathway [62]. This has led us to assume that while the reduction of pAKT was necessary for a synergistic effect of the inhibitors of COX-2 and CYP19A1, the activation of ERK may affect the magnitude of the synergistic effect, strong or moderate based on the increased or decreased activation of ERK, respectively. Overall, the results indicate pAKT as a critical pathway in the interaction between COX-2 and CYP19A1 forming a positive loop that fuels the pathogenesis of MPM. In light of this, COX2-CYP19A1 axis could be pharmacologically targeted opening up new therapeutic options. Therefore, future in vivo studies to evaluate the effect of the combination of rofecoxib and exemestane are warranted. Several lines of evidence indicate that PI3-K/Akt survival pathway is involved in the regulation of COX-2 and PGE2 synthesis in human cancer [63–66]. In breast tumors, the association between COX-2 expression and AKT phosphorylation, which was a poor outcome marker, was demonstrated in PGE2’s ability to induce AKT phosphorylation in the estrogen negative receptor [67]. Since PGE2 in MPM increased the expression and level of CYP19A1, we assayed in a 3D MPM spheroid model the effect of COX-2 and CYP19A1 inhibitors alone or in combination, have on the COX-2-induced CYP19A1 activity. In a preclinical setting, we employed the 3D tumor spheroid model that mimics the behavior of cancer cells found in solid tumors in vivo [68]. Single treatment with rofecoxib or exemestane or their combination caused a marked reduction in sphere-forming efficiency and size especially in the Ist Mes1 and MPP89 spheroids, suggesting that the treatments is effective in this proxy preclinical models. Furthermore, IHC analysis of COX-2 and E2, the product of CYP19A1 activity, on MPP89 spheroids after treatment with rofecoxib or exemestane or their combination resulted in a reduction of COX-2 and E2 staining, indicating that COX-2 inhibition acted on the CYP19A1 product. Moreover, beyond the E2 staining, exemestane also the COX-2 staining, suggesting that there was an interplay between E2 and COX-2 that was worth investigating. In light of this, to investigate whether these observations could have some clinical relevance, we decided to analyze E2 and COX-2 expression in archival human MPM samples. We found that COX-2 and E2 was expressed in the majority of MPM samples as a cytoplasmic protein. IRS, commonly utilized in both the clinical setting and translational research, was also used in the present study to establish a reliable semi-quantitative scoring system that takes into account the percentage of stained cells as well as the intensity of staining [69]. E2 IRS was mainly moderate and COX-2 IRS was mainly mild. Of note, in MPM spheres the IRS of E2 was higher than the IRS of COX-2 indicating that the data obtained on the spheroids was confirmed in the tissue samples. Moreover, in the MPM specimens that showed high score value for COX-2 and E2 expression, a direct correlation between COX-2 and E2 was demonstrated.

## Conclusions

**Fig. 7:**
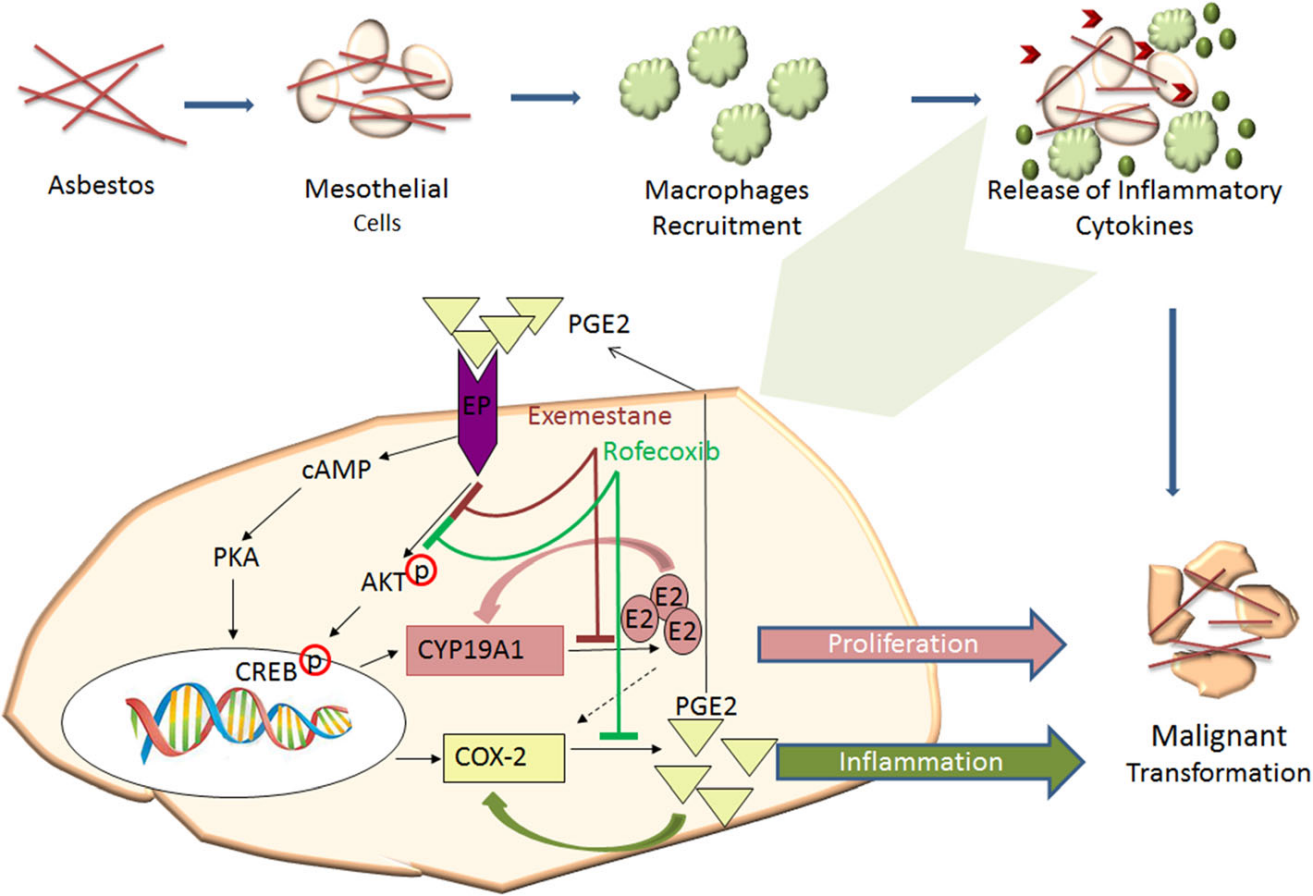
Schematic representation of the interplay of COX-2 and CYP19A1 in the asbestos cancerogenesis. Upon inhalation of asbestos fibers, mesothelial and inflammatory cells release a variety of cytokines that inhibit asbestos-induced cytotoxicity and induce inflammation. PGE2 is abundantly produced in the region of inflammation. PGE2-mediated activation of EP receptors leads to enhanced the cAMP/PKA and phosphatidylinositol 3-kinase (PI3K) signaling pathways. increased in cyclic adenosine monophosphate (cAMP) production activates protein kinase A (PKA) -CREB dependent expression of genes including COX-2 and CYP19A1. AKT activation (pAKT) induces the CREB activation (pCREB) resulting in increased CYP19A1 and COX-2 transcription and activities. This lead to enhanced E2 and PGE2 biosynthesis. The local concentrations of E2 upregulates CYP19A1 and COX-2 and PGE2 induces CYP19A1 and COX-2 and establishes a positive-feedback loop in favor of continuous E2 and PGE2 formation that results in increased proliferation of tumor cells and inflammation that ultimately leads to the onset of MPM. The combined use of rofecoxib and exemestane by reducing the levels of PGE2 and E2, respectively and the activation of AKT (pAKT) reduces inflammation and cell proliferation

These observations strongly highlighted an interplay between COX-2 and CYP19A1 activity in the pathogenesis of MPM and suggested a further mechanism by which asbestos inflammation could cause the onset of MPM (Fig. 7). Interestingly, the reduction of AKT activation induced by the combination of COX-2 and CYP19A1 inhibitors in MPM cells represents a critical pathway in the response to treatment as detected in the onset of drug resistance in a variety of cancers [70]. Therefore, the association of COX-2 and CYP19A1 inhibitors could be further investigated for therapeutic purposes in subjects who manifest pleural pathologies after exposure to asbestos.

## Supplementary Information

**Additional file 1: Supplementary Table 1.** Immunostaining of COX-2 and CYP19A1 in MPM samples.

**Additional file 2: Supplementary Figure 1. **The graphs represent the mean ± SD of three independent quantifications of protein band intensities normalized first compared to the load control (vinculin or tubulin) and then in comparison to the untreated sample (relative band intensity) from western blots of cell cycle and apoptosis protein expression in Ist Mes1, Ist Mes2 and MPP89 cells treated with EXE, ROF and EXE+ROF for 48 h.*, statistically significant effects (paired Student t test *P* < 0.05) compared to CNTR.

## Data Availability

Not applicable.

## Abbreviations

MPM: Malignant pleural mesothelioma
COX-2: Cyclooxygenase-2
PGE2: Prostaglandin E2
CYP19A1: Aromatase
E2: Estradiol
PI3K: Phosphoinositide 3-kinase
ERK1/2: Extracellular Signal–Regulated Kinase 2
CI: Combination index
FACS: Fluorescence-activated cell sorting
RT-PCR: Reverse transcriptase-polymerase chain reaction
IHC: Immunohistochemical analysis
IRS: Immunoreactivity score
