# Correction: Identification of novel COX-2 / CYP19A1 axis involved in the mesothelioma pathogenesis opens new therapeutic opportunities

**DOI:** 10.1186/s13046-022-02539-3

**Published:** 2023-01-11

**Authors:** Barbara Nuvoli, Barbara Antoniani, Roberta Libener, Antonio Maconi, Andrea Sacconi, Mariantonia Carosi, Rossella Galati

**Affiliations:** 1 Preclinical Models and New Therapeutic Agents Unit, IRCCS Regina Elena National Cancer Institute, Rome, Italy; 2 Anatomy Pathology Unit, IRCCS Regina Elena National Cancer Institute, Rome, Italy; 3 Department of Integrated Activities Research and Innovation, SS Antonio and Biagio General Hospital, Alessandria, Italy; 4 Clinical Trial Center, Biostatistics and Bioinformatics Unit, IRCCS Regina Elena National Cancer Institute, Rome, Italy

**Correction:** ***J ExpClin Cancer Res*** **40, 257 (2021)**

**https://doi.org/10.1186/s13046-021-02050-1**

Following publication of the original article [1], author identified an error in Fig. 4a and b, specifically:

- Figure 4a - Vinculin in MPP89
- Figure 4b - Tubulin Ist Mes2 and MPP89

**Fig. 4:**
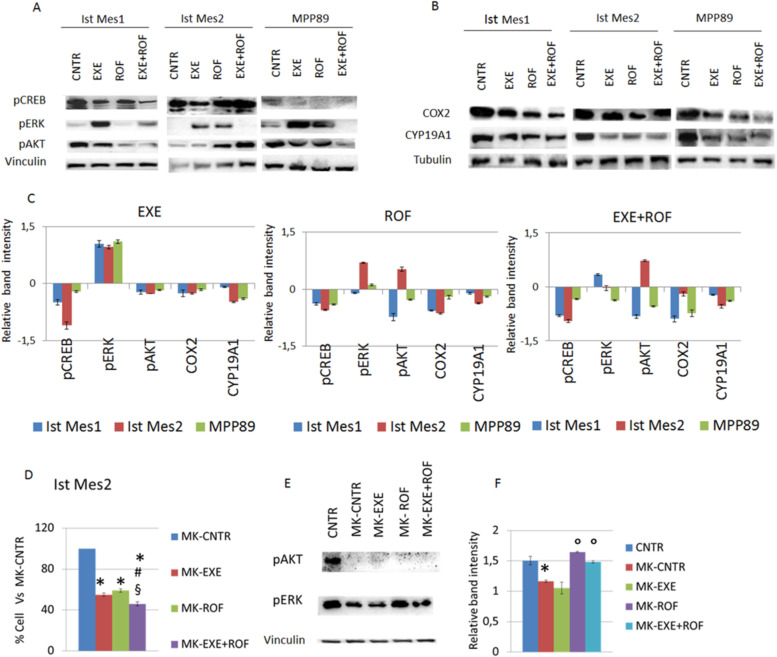
AKT and ERK phosphorylation are implicated in the combined action of rofecoxib and exemestane. **A** Representative experiment out of three independent western blot analyses of pCREB, pERK and pAKT expression in Ist Mes1, Ist Mes2 and MPP89 cells treated with 35μM exemestane (EXE) or 35μM rofecoxib (ROF) or 35μM exemestane and 35μM rofecoxib combination (EXE + ROF) for 30 min. **B** Representative western blot analyses of COX-2 and CYP19A1 expression in Ist Mes1, Ist Mes2 and MPP89 cells treated with 35μM exemestane (EXE) or 35μM rofecoxib (ROF) or 35μM exemestane and 35μM rofecoxib combination (EXE + ROF) for 24 h. **C** The graphs represent the mean ± SD of three independent quantifications of protein band intensities normalized to the loading control and then in comparison to the untreated sample (relative band intensity). **D** The graph represents the mean ± SD of three independent cell survival rates after pre-incubation with MK-2206 and subsequently treated with exemestane (MK-EXE), or rofecoxib (MK + ROF) or exemestane and rofecoxib combination (MK-EXE + ROF) compared to untreated (100 % of cell alive). **E** Representative western blot analyses of pAKT and pERK expression in Ist Mes2 cells pre-incubated with MK-2206 and after treated with exemestane (MK-EXE), or rofecoxib (MK + ROF) or exemestane and rofecoxib combination (MK-EXE + ROF). **F** The graph represents the mean ± SD of three independent quantifications of protein band intensities normalized to the loading control. Statistically significant effects (paired Student t test *P* < 0.05) compared to CNTR *, EXE # or ROF § or MK-CNTR °

The correct figure is presented below:

This correction does not change the result, interpretation, and conclusions of the study. The original article has been corrected.
