# Supplementary material for: Identification of novel COX-2 / CYP19A1 axis involved in the mesothelioma pathogenesis opens new therapeutic opportunities
Source: J Exp Clin Cancer Res. 2021 Aug 17;40:257. doi: 10.1186/s13046-021-02050-1 (PMC8369782; doi:10.1186/s13046-021-02050-1)
Supplement: Supplementary file 1 — Additional file 1: Supplementary Table 1. Immunostaining of COX-2 and CYP19A1 in MPM samples. [file 13046_2021_2050_MOESM1_ESM.docx]

**Supplementary Table 1** Immunostaining of COX-2 and CYP19A1 in MPM samples

| Patients | Hystology | Score  CYP19A1  0=absent  1=low  2=high | Score  CYP19A1  0=absent  1=low  2=high |
| --- | --- | --- | --- |
| 1 | Epithelial | 1 | 1 |
| 2 | Epithelial | 0 | 1 |
| 3 | Epithelial | 2 | 2 |
| 4 | Epithelial | 2 | 2 |
| 5 | Epithelial | 1 | 1 |
| 6 | Epithelial | 2 | 2 |
| 7 | Epithelial | 0 | 1 |
| 8 | Epithelial | 2 | 2 |
| 9 | Epithelial | 2 | 2 |
| 10 | Epithelial | 1 | 1 |
| 11 | Epithelial | 0 | 1 |
| 12 | Epithelial | 2 | 2 |
| 13 | Epithelial | 2 | 2 |
| 14 | Epithelial | 2 | 2 |
| 15 | Epithelial | 2 | 1 |
| 16 | Epithelial | 1 | 1 |
| 17 | Sarcomatoid | 0 | 1 |
| 18 | Sarcomatoid | 2 | 2 |
| 19 | Sarcomatoid | 2 | 2 |
| 20 | Sarcomatoid | 2 | 2 |
| 21 | Sarcomatoid | 2 | 2 |
| 22 | Sarcomatoid | 0 | 1 |
| 23 | Biphasic | 2 | 2 |
| 24 | Biphasic | 2 | 2 |
| 25 | Biphasic | 2 | 2 |
| 26 | Biphasic | 2 | 2 |
| 27 | Biphasic | 2 | 2 |
| 28 | Biphasic | 2 | 2 |
| 29 | Biphasic | 2 | 2 |
