# Supplementary material for: Identification of novel COX-2 / CYP19A1 axis involved in the mesothelioma pathogenesis opens new therapeutic opportunities
Source: J Exp Clin Cancer Res. 2021 Aug 17;40:257. doi: 10.1186/s13046-021-02050-1 (PMC8369782; doi:10.1186/s13046-021-02050-1)
Supplement: Supplementary file 2 — Additional file 2: Supplementary Figure 1. The graphs represent the mean ± SD of three independent quantifications of protein band intensities normalized first compared to the load control (vinculin or tubulin) and then in comparison to the untreated sample (relative band intensity) from western blots of cell cycle and apoptosis protein expression in Ist Mes1, Ist Mes2 and MPP89 cells treated with EXE, ROF and EXE+ROF for 48 h.*, statistically significant effects (paired Student t test P < 0.05) compared to CNTR. [file 13046_2021_2050_MOESM2_ESM.docx]

**Supplementary Figure 1.** The graphs represent the mean ± SD of three independent quantifications of protein band intensities normalized first compared to the load control (vinculin or tubulin) and then in comparison to the untreated sample (relative band intensity) from western blots of cell cycle and apoptosis protein expression in Ist Mes1, Ist Mes2 and MPP89 cells treated with EXE, ROF and EXE+ROF for 48 h.*, statistically significant effects (paired Student t test P < 0.05) compared to CNTR.

**Supplementary Figure 1**

**
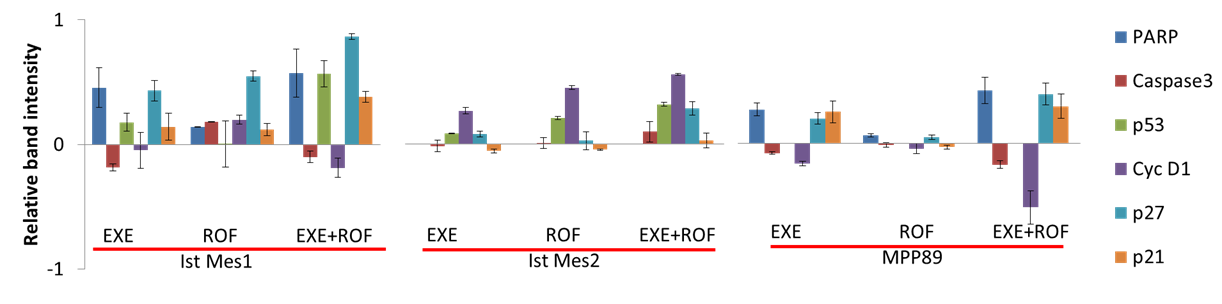
**
